# Placental malaria infection is associated with downregulation of STAT-6 and ANG-1 in decidual macrophages

**DOI:** 10.3389/fimmu.2025.1497936

**Published:** 2025-02-11

**Authors:** Fred Owino, Caroline Kijogi, Omu Anzala, Edwin Walong, Obiero Jael, Steven G. Nyanjom, Agola Lelo Eric, Bernard N. Kanoi, Jesse Gitaka

**Affiliations:** ^1^ Biochemistry Department, Jomo Kenyatta University of Agriculture and Technology, Juja, Kenya; ^2^ Centre for Biotechnology Research and Development, Kenya Medical Research Institute, Nairobi, Kenya; ^3^ Centre for Malaria Elimination, Institute of Tropical Medicine, Mount Kenya University, Thika, Kenya; ^4^ Centre for Research in Infectious Diseases, Mount Kenya University, Thika, Kenya; ^5^ Department of Pharmacotherapy of Lifestyle-related Diseases, Graduate School of Pharmaceutical Sciences, Tohoku University, Sendai, Japan; ^6^ Department of Medical Microbiology, Kenya AIDS Vaccine Initiative (KAVI) Institute of Clinical Research, Nairobi, Kenya; ^7^ Department of Medical Microbiology, University of Nairobi, Nairobi, Kenya; ^8^ School of Medicine, Maseno University, Kisumu, Kenya; ^9^ Reproductive Health and Biology, Kenya Institute of Primate Research, Nairobi, Kenya

**Keywords:** placental malaria, macrophages, M1/M2 macrophage polarization, transcription factors, angiogenic factors

## Abstract

**Introduction:**

Macrophages play a crucial immunological role in maintaining pregnancy. Placental malaria infection may cause dysfunction in decidual macrophages which then culminates in the associated pregnancy complications. Here, we determined the influence of placental malaria on decidual macrophages, by assessing their distribution based on their unique phenotypes, and examining their expression levels of transcription factors as well as angiogenic factors, in placentas from women living in a malaria-endemic area.

**Methods:**

We compared these macrophage parameters in placentas from malaria infected women to those from the uninfected women. Placentas were collected upon delivery and malaria infection determined by histology together with PCR from dry blood spots obtained from placental blood. Following enzymatic dissociation of placental tissue, immune cells were enriched from the total population of placental cells by density centrifugation. Macrophage phenotypic characteristics were then analyzed from the placental immune cells by flow cytometry. The expression of surface markers CD68, CD80, CD86, CD163, CD206, and CD209, was used to delineate the macrophage populations. For gene expression profiling, macrophages were isolated from the placental immune cells and the expression level of transcription factors *STAT-1, IRF-5, STAT-6, c-Maf* and angiogenic factors *ANG-1, ANG-2* and *VEGF* determined by qPCR.

**Results and Discussion:**

We found no difference in the total macrophage populations and M1 and M2 macrophage profiles between uninfected and infected placentas, however, M2 macrophages were significantly higher compared to their M1 counterparts regardless of infection status. Notably, the gene expression levels of the transcription factor *STAT-6* and angiogenic factor *ANG-1* were significantly lower in infected placentas. These findings provide a basis for further understanding of the role of placental macrophages in placental malaria pathogenesis. Analysis of the functional consequences of these observations is needed to determine if these factors can be explored to reprogram macrophage polarization to desired state.

## Introduction

From a global perspective, it is estimated that close to half of the population in the world is at risk of malaria infection with the greatest burden of malaria-related deaths occurring in the WHO African Region including Kenya. Children and pregnant women are at the highest risk for morbidity and mortality particularly in areas of stable malaria transmission (1). In pregnancy, malaria infection is associated with poor maternal and fetal outcomes such as maternal anemia, preterm delivery, intrauterine growth restriction, low birth weight, miscarriage, and stillbirth (2). Peripheral blood infection by malaria parasite may lead to cytoadherence of the parasite in internal organs whereby malaria-infected red blood cells accumulate in the microvasculature. In pregnant women this can occur in the placenta and the malaria parasite *Plasmodium falciparum* is the main culprit of this sequestration in the placental intervillous spaces causing placental malaria (PM) (3).

PM triggers inflammatory responses characterized by the infiltration of immune cells, mainly monocytes and macrophages (4, 5) subsequently disrupting the pro-inflammatory and anti-inflammatory immune balance required for a healthy pregnancy. While this infiltrate may help to control parasite replication, it also leads to impaired placental development and function, through the mechanical compromise of placental circulation and a dysregulated production of inflammatory mediators caused by the massive inflammation (6). Though several studies have investigated these immunological changes due to PM by assessing the functional response (production of cytokines and chemokines) (7–9), few studies have dissected the cellular profile of the placental macrophage infiltrates. These cells represent unique effectors of local immunity in the placenta capable of either a pro-inflammatory (M1 macrophages) function or an anti-inflammatory (M2 macrophages) function depending on their polarization and activation status. While the M1/M2 classification of macrophages remains a matter of debate due to the extensive state of macrophage subpopulations associated with intermediate or incomplete polarization (10), this classical concept is still useful for our understanding of placental malaria-related immunopathology given the limited knowledge regarding macrophage rewiring in the context of malaria infection.

During the early phase of a healthy pregnancy, the immune cells in the placenta consisting predominantly of uterine NK (uNK) cells and macrophages, play a critical role in the maintenance of maternal-fetal tolerance. Whereas uNK cells undergo massive apoptosis later in pregnancy and are virtually absent at term, macrophages are present in the decidua throughout till the end of the pregnancy (11, 12). These decidual macrophages have been characterized as mainly anti-inflammatory and regulatory (M2-like phenotype), typical for the prevention of fetus rejection (13). However, in pathological pregnancy such as that of PM and other pregnancy-associated diseases, conflicting reports exist on pro- and anti-inflammatory polarization of placental macrophages (14–17). Considering the potential loss of immunotolerance against the fetus and a heightened inflammatory process, the investigation of the phenotypic distribution and gene expression pattern of placental macrophages in the context of PM is vital to better elucidate the pathophysiology of the disease and possibly contribute to future therapy strategies.

The goal of this study therefore was to investigate the effect of PM on maternal macrophages by quantifying M1 and M2 macrophages using previously described delineating surface marker expression (18), and by examining the gene expression profiles of key transcription factors, relevant for macrophage polarization (19), as well as angiogenic factors, important for maintenance of a competent vascular network [ (20, 21), in the placenta of pregnant women. Comparable distribution of M1 and M2 macrophages was observed by surface marker expression between infected and uninfected placentas. However, the gene expression levels of the M2 transcription factor, *STAT-6* and the angiogenic factors *ANG-1*, were significantly lower in the placenta from malaria-positive women.

## Materials and methods

### Study design

**Figure 1:**
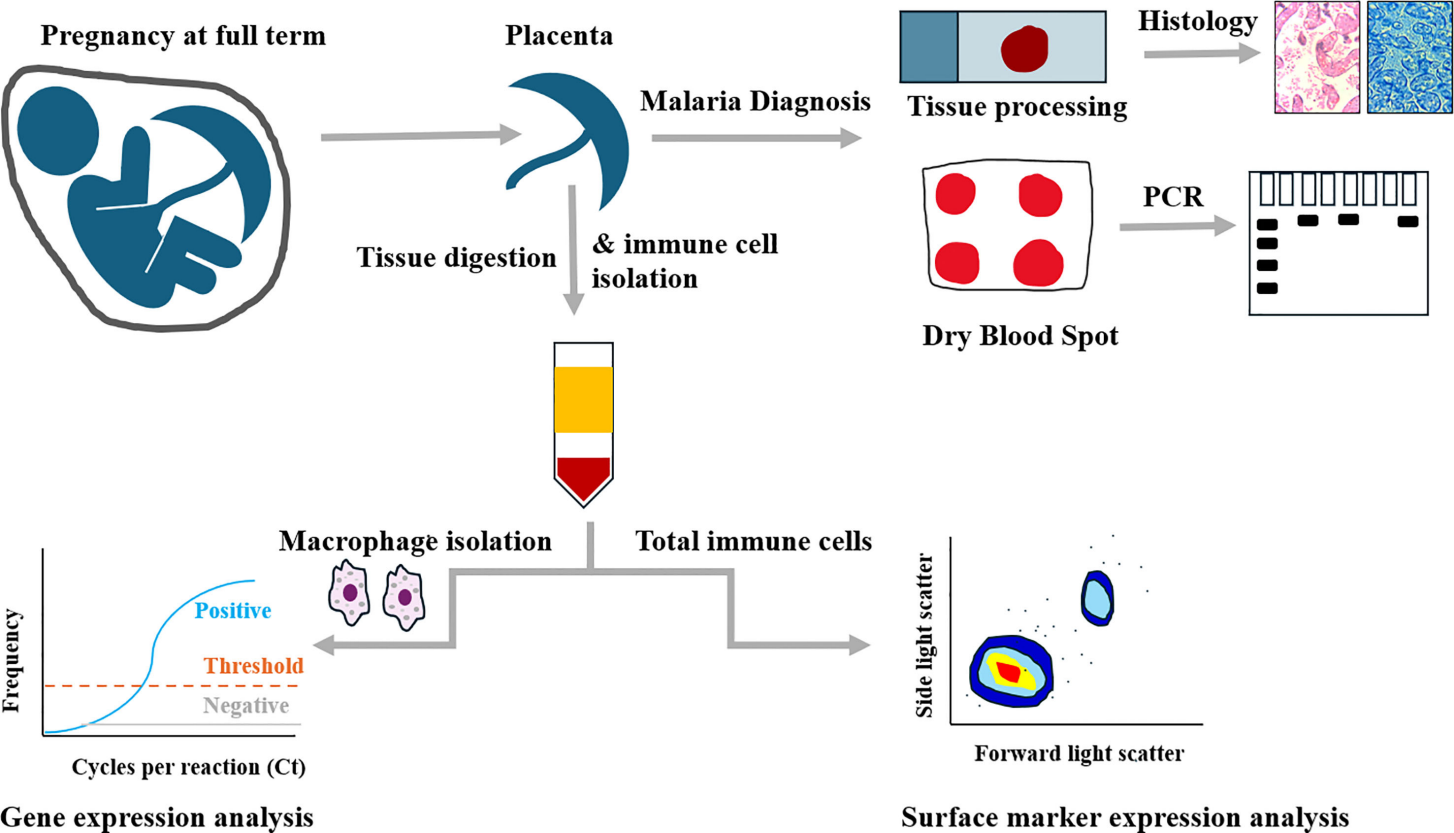
Graphical abstract of experimental design.

The 66 samples used in this study were collected from pregnant mothers with term deliveries of uncomplicated, singleton pregnancies attending Webuye County Hospital in Bungoma, western Kenya, in 2021. Participants were recruited during their antenatal clinic visits and upon explanation of study activities, those who provided written informed consent were enrolled. Inclusion criteria included women aged between 18 to 45 years with no known comorbidities and having antenatal records. Exclusion criteria included gravida ≥ 4, reported hypertension, diabetes, HIV infection, and history of long-term antibiotic treatment. Before delivery, a structured questionnaire was administered to collect demographic data while the clinical data was retrieved from the antenatal book records. Participant characteristics are summarized on **Table 1** **. **A graphical abstract of the experimental design is shown in **Figure 1**. Ethical approval for the study was obtained both from the institutional scientific and ethics committees of Mount Kenya University (MKU/ISERC/684 and MKU/ISERC/2822) and the Kenya Medical Research Institute (KEMRI/SERU/CBRD/214/4118).

**Table 1:** Characteristics of study participants.

Parameters: median (range)		All women (n = 66)	Histology PM - women (n = 33)	Histology PM + women (n = 26)	P value	Placental Blood PCR PM-women (n = 31)	Placental Blood PCR PM + women (n = 27)	P value
**Age (years)**		23 (18–37)	25 (18–37)	21.5 (18–37)	0.758	26 (18–37)	23 (18–32)	0.0370
Primigravid		26 (39%)	15 (58%)	11(42%)		11 (61%)	7 (39%)	
Secundigravid		27 (41%)	14 (70%)	6 (30%)		10 (37%)	17 (63%)	
Multigravid		13 (20%)	4 (31%)	9 (69%)		10 (77%)	3 (23%)	
**Gestational age (wks)**		38.6 (29–44)	38.6 (29–43)	39 (31.4–44)	0.264	38.6 (31–41.3)	38.6 (27–44)	0.8850
**Baby birth weight (Kg)**		3.2 (1.7–5.0)	3.5 (1.7–5.0)	3.25 (2.0–4.5)	0.303	3.2 (1.7–4.5)	3.4 (2.2–5.0)	0.6470
**Hemoglobin Level (g/dL)**		11.6 (7.2–15.1)	11.5 (7.5–14)	10.8 (7.2–15.1)	0.862	11.15 (7.5–14.1)	12.4 (7.2–15.1)	0.1660
**iPTP Dose**		3 (0–8)	4 (1–8)	4 (0–7)	0.531	4 (0–8)	4 (1–7)	0.7550
UTI in Pregnancy		15/62 (24%)	3 (23%)	10 (77%)		7 (50%)	7 (50%)	

In bold: Participant characteristics whose statistical parameters were determined.

### Placenta tissue sampling

**Figure 2:**
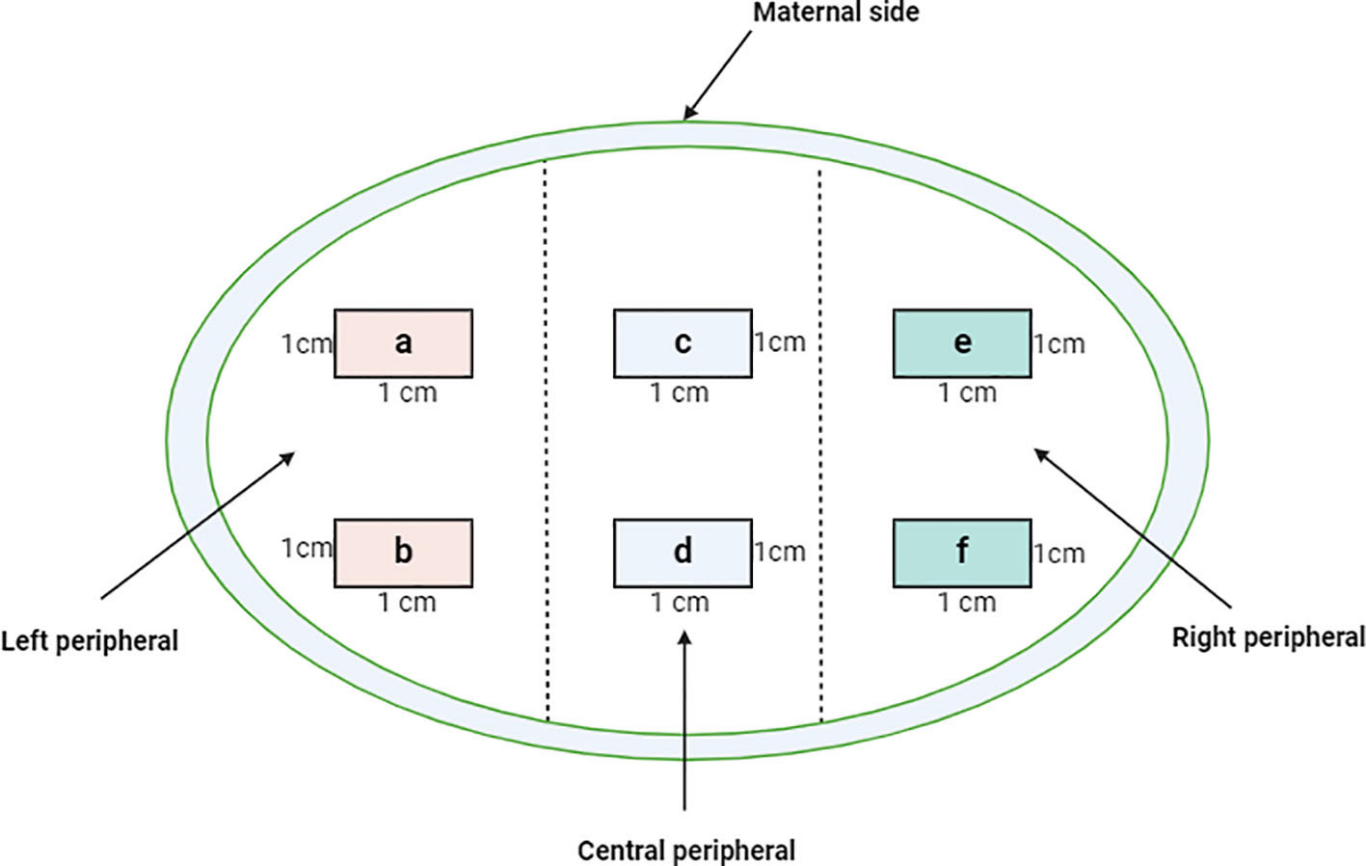
Cross sectional representation of the maternal placenta indicating the sampling criteria of the three quadrats from which the six tissue blocks **(A-F)** were obtained. 1gm tissue from the blocks was excised and used for varying downstream analyses such as flow cytometry and gene expression profiling which were done simultaneously. The tissues were minced and enzymatically digested to retrieve total immune cells. For histology, a single slide from each of the blocks was used and the results harmonized.

Placental samples were collected within two hours after delivery. To provide a reasonable representation of the tissue, we adopted the sampling approach as reported by Roberts et al. (22) with modifications. Briefly, from the maternal side of the placenta, incisions were extended to the fetal side without reaching the fetal membrane. The incisions were made on three equidistant sections on the longest axis of the placenta (that includes the insertion of the umbilical cord); left, center, and right sections. Based on the sections, 1cm on either side of the cord insertion point and 1cm from the periphery of the placenta was collected as illustrated in the schematic in **Figure 2**. Six 1cm^3^ pieces per section were collected, snap-frozen, and stored at -80°C until further processing. For tissue fixation, mirrored excised tissues were collected in 10% neutral buffered formalin (NBF) that offers a stable pH level essential for maintaining tissue integrity and prevents the formation of formalin pigment.

### Tissue processing for histopathological malaria diagnosis

**Figure 3:**
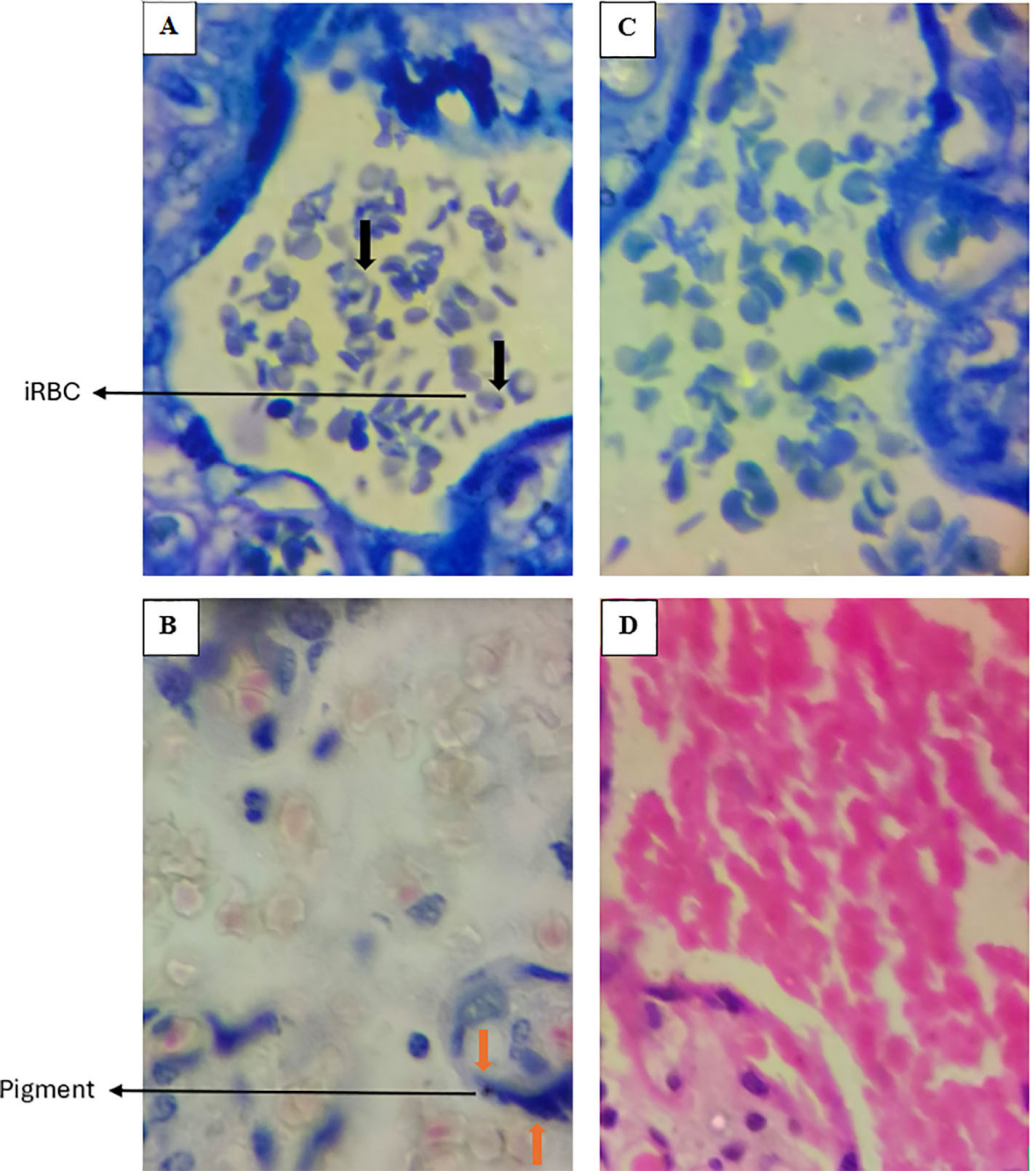
Representative images of placentas stained with Giemsa (Upper deck) and H&E (Lower deck), demonstrating histological evidence of *P. falciparum* infection in panels **(A, B)** Uninfected placentas are shown in panels **(C, D)** Black arrows indicate infected erythrocytes (iRBC), while orange arrows highlight the presence of pigment. In the uninfected placentas **(C, D)**, there are no infected RBCs nor presence of pigment. Images were taken at 100X magnification.

Portions of the fixed tissues were grossed and placed into a well-labeled tissue processing cassette and further fixed in NBF for about an hour. The tissue portions in the cassette were dehydrated in ascending grades of ethanol (60%, 70%, 80% and 90%) for 90 minutes. They were then placed in three changes of absolute ethanol to achieve full dehydration. After dehydration, the tissues were cleared in two changes of xylene and transferred into molten paraffin wax at a melting temperature of 56°C. Infiltration was carried out for 2 hours. For paraffin embedding, an embedder (GST-32AGOPC6172F1ZL, IndiaMART, Kerala, India) was used to form the tissue blocks. The blocks were trimmed and 5µm thick of tissues were sectioned and mounted onto well labeled glass slides. Subsequently, the slides were air dried, heat fixed and stained using standard hematoxylin and eosin (H&E) stains and Giemsa. The sections were examined under a light microscope for detection of trophozoites or parasitized erythrocyte, hemozoin, pigmentation, knotting, and vasculitis among other defining parameters as previously described (23, 24). Placental malaria was defined as the presence of *P. falciparum* parasite (trophozoite), malaria pigment, or the presence of hemozoin in the intervillous space. Representative placental images depicting the stained sections, and the observed infection status are shown in **Figure 3**.

### Malaria diagnosis by PCR

DBS samples were prepared by spotting a drop of whole placental blood onto Whatman filter papers (GE/Whatman, Kent, UK), air-dried for at least six hours, preserved individually in clean zippered plastic bags with silica gel, and stored at -80°C freezer until DNA extraction. Three uniform-sized punches of approximately 6mm diameter were excised from the dried blood spots and total DNA was extracted using QIAamp DNA Mini Kit (QIAgen, Hilden, GERMANY) following the manufacturer’s instructions. For greater detection sensitivity, nested PCR for the *Plasmodium* mitochondrial cytochrome c oxidase III (cox3) gene was performed as described by Isozumi et al. (25). Briefly, the primary PCR was carried out in a 25µL reaction containing 5µL of template DNA, 0.4mM plasmodium-genus specific primer set and 12.5 µL of the GoTaq Green Master Mix (Promega, Madison, WI, USA). Cycling conditions comprised of an initial activation at 94°C for 2 min, followed by 35 cycles at 94°C for 20 sec, 63°C for 1 min 30 sec, and a final extension step at 63°C for 5 min. The primary PCR product was diluted 1:50 with sterile water and used as a template for the secondary PCR. The reaction mixture for the secondary PCR consisted of a 25µL volume containing 1µL of the diluted PCR product, 0.4uM falciparum species-specific primer set, and 12.5 µL of the GoTaq Green Master Mix. Cycling conditions comprised of an initial denaturation at 95°C for 2 min, followed by 25 cycles at 95°C for 20 sec, 56°C for 1 min 30 sec, and a final extension at 56°C for 5 min. All amplifications were performed on the Applied Biosystems MiniAmp Plus Thermal Cycler (Thermo Fisher Scientific, Waltham, MA, USA). Amplification products were analyzed by electrophoresis in 2% agarose gel.

### Isolation of placental leukocytes

Maternal leukocytes were isolated from the tissue using previously described protocols (26, 27) with modifications. Briefly, the six tissue blocks previously sampled and stored, three from each quadrat were thawed on ice and washed with 1X PBS (phosphate-buffered saline). From each block of tissue, approximately 1-gram plugs were excised using a scalpel. The tissues were then placed on petri dish, cut and minced as possible. The minced tissue was enzymatically digested at 37°C with gentle agitation in 2 series of digestion solutions containing; RPMI 1640 media supplemented with 1% penicillin-streptomycin and 1M HEPES buffer (R0), 1% 12.5mM DNase DNase I, and 10mg/mL collagenase II (SIGMA). The cell suspension was filtered through 100 mm strainers into 15mL tubes containing FBS, pelleted, resuspended in R0 containing 10% FBS (R10), layered onto 5mL of histopaque, centrifuged at 1000Xg for 20 min without brake and acceleration to collect mononuclear cells. A proportion of the cells were assayed for cell surface markers and from the rest of the cells, macrophages were isolated for downstream application.

### Immunophenotyping

Total cell counts were performed prior to staining. The isolated cells were stained with various surface markers, at 1:50 or 1:100 antibody concentrations, in 1XPBS for 30 min at 4°C protecting from light. BD^®^ CompBeads were used for single-color compensation controls. The antibodies included CD68-PE/BV421, CD80-PECy5/PECy7, CD86-APC/PECy5, CD206-PECy7, CD209-FITC, CD163-FITC. In this study, CD68 was applied as a pan macrophage marker (28–30) and CD80 and CD86 as markers for M1-like polarized macrophages. CD206 and CD209 or CD163 were utilized to the M2-like sub-population (14, 17, 31, 32). After staining, the cells were washed with PBS to remove excess antibody resuspended in 0.3 ml stain PBS and acquired using the BD LSR II Flow Cytometer (BD Biosciences) and BD FACSDiva 6.0 software (BD Biosciences). Data obtained was analyzed by FlowJo version 10. During cell gating the cells were initially selected by size using FCS and SSC. The gating strategy included the exclusion of doublets and dead cells, and accounting for specific signal by using unstained samples and FMOs as negative control for each antibody.

### Isolation of macrophages and quantitative RT-PCR

Macrophages were isolated from the remaining portion of the isolated mononuclear cells by positive selection using Dynabeads precoated with anti-CD14 antibody (Invitrogen) following manufacturer’s instructions. The cells were then lysed directly, and total RNA extracted using RNeasy kit (QIAGEN, Hilden Germany) as per standard manufacturer’s protocol. The integrity of the purified RNA was assessed by spectrometry. To investigate expression levels of the target genes (*STAT-1, IRF-5, STAT-6, c-Maf, ANG-1. ANG-2*, and *VEGF*), purified RNA samples were first converted to cDNA by the heat-sensitive FastKing gDNA dispelling RT Supermix kit (Tiagen), and qRT-PCR performed using primers for the selected targets (**Supplementary Table S1**) and Evagreen HRM analysis kit (Tiagen) on the Rotor gene 2plex Real-time PCR cycler (QIAGEN, Rotor, Hilden, Germany). The amplifications were carried out for 42 cycles (10 sec at 95°C and 20 sec at 60°C and 30 secs at 72°C) and the melt was done at 72°C. At least 3 technical replicates were analyzed for all comparisons. Differential expression was calculated via the ΔΔCT method (33). Expression was normalized using the mean CT of the housekeeping gene, *GAPDH*.

### Statistical analysis

The statistical analyses were conducted using GraphPad Prism version 9.5.1 (GraphPad Software, San Diego, CA) and R software package version 4.1.3. Data was first tested for normal distribution using Kolmogorov-Smirnov test ahead of the comparisons. For pairwise comparisons, normally distributed data was compared by t-test, while for data not following a normal distribution the Mann-Whitney U test was used. For multiple comparisons, ANOVA and Kruskal-Wallis tests were utilized for normally and non-normally distributed data respectively. Multivariate analysis was employed to adjust for potential confounders, including age, gravidity and IPTp usage. Findings with p-values < 0.05 were considered significant.

## Results

### Characteristics of the study participants

The placental infection was investigated by histology and PCR. Selection of samples from the sixty-six participants was based on the quality of prepared sections for histology and quality of DBS for PCR. Evidence of PM was observed in 26 (44.1%) and 27 (46.6%) by histopathology and PCR respectively (**Table 1**). Malaria had no influence on gravidity, term of delivery, birth weight and hemoglobin levels, both when stratified by histopathology and PCR. However, when stratification of infection status was done by PCR only, infected mothers were significantly younger (P=0.037). Most women received intermittent preventive therapy (IPTp), which has previously been shown to significantly reduce maternal and placental infections (34, 35). The average dose of IPTp received in malaria-infected pregnant women did not differ from that of malaria-uninfected women (**Table 1**). Nevertheless, stratification by histopathology revealed that malaria infection was associated with urinary tract infection (UTI) (**Table 1**). It is plausible that an increase in systematic inflammatory burden orchestrated by UTIs could increase the risk of contracting malaria.

### Quantification of M1 and M1 macrophages by flow cytometry

**Figure 4:**
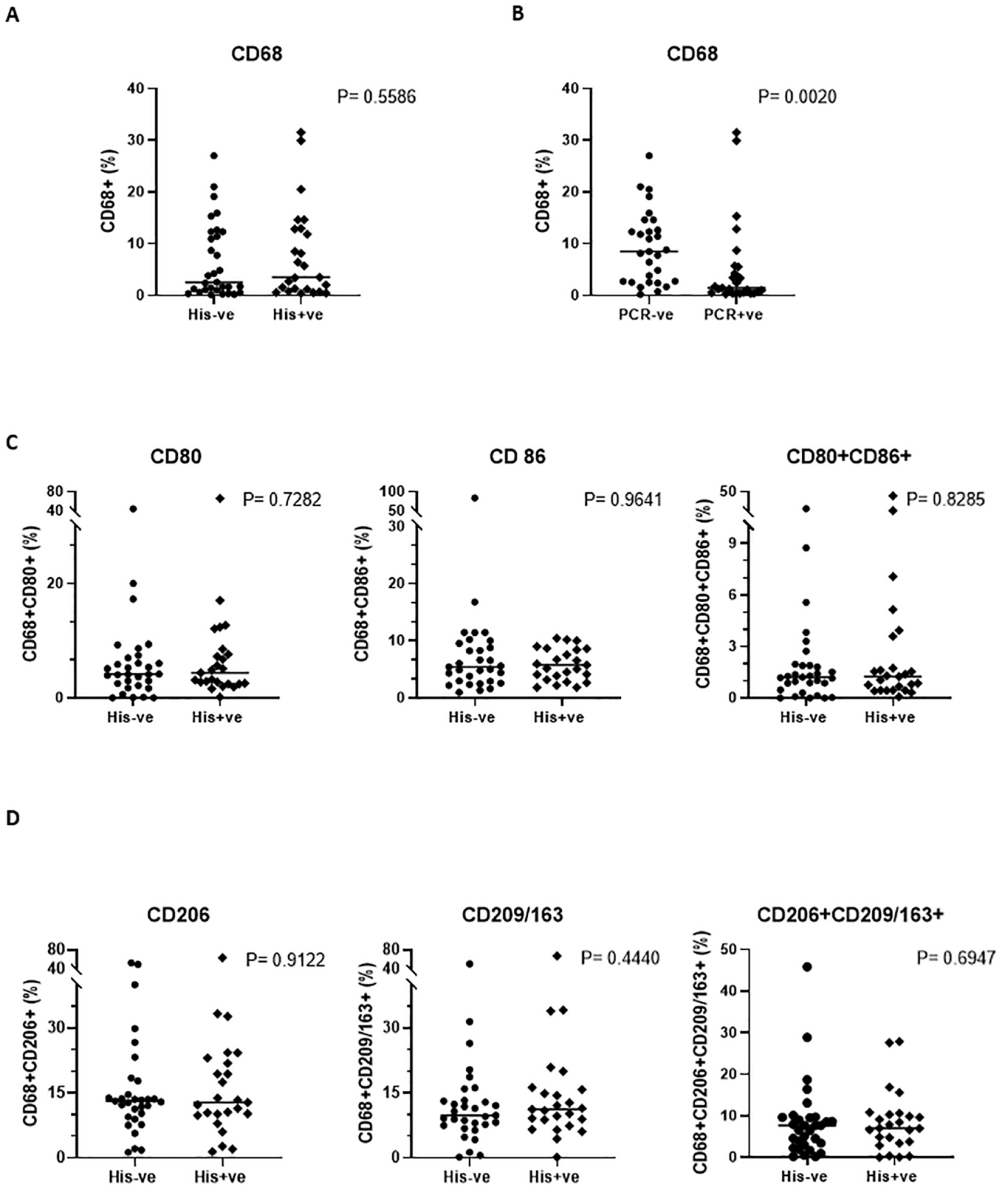
Phenotypic profile of the placental macrophage subsets. A comparison of the general macrophage population (CD68) when stratification is by histology **(A)** (P=0.5586) and by PCR **(B)** (P=0.0020) is shown, while subset specific distribution comparison is depicted in **(C, D)**. Mann-Whitney U and student’s t tests were used for evaluating differences among the two groups.

To assess macrophage polarization in the placenta, we examined the distribution of macrophage populations by flow cytometric analysis of the isolated total immune cells. The gating strategy is illustrated in **Supplementary Figure S1**. We defined CD68+CD80+ and CD68+CD86+ cells as M1 macrophages, and CD68+CD206+ and CD68+CD209+ or CD68+CD163+ cells as M2 macrophages). There was no significant difference in the proportion of total macrophages, regardless of subtype (CD68+ only), between placentas from uninfected and infected women (**Figure 4A**). However, when infection was stratified by PCR total macrophages markedly decreased in placentas from women with malaria (**Figure 4B**). Decrease in the number of macrophages has also been observed in other placental pathologies involving infections (36, 37). For the targeted macrophage populations, the frequencies of both M1 macrophages, (**Figure 4C**) and M2 macrophages, (**Figure 4D**), did not show any difference in their distribution between the two groups but when compared against each other, M2 macrophages were significantly higher than their M1 counterparts in general (**Supplementary Figures S2**).

### Analysis of gene expression in decidual macrophages by RT-PCR

**Figure 5:**
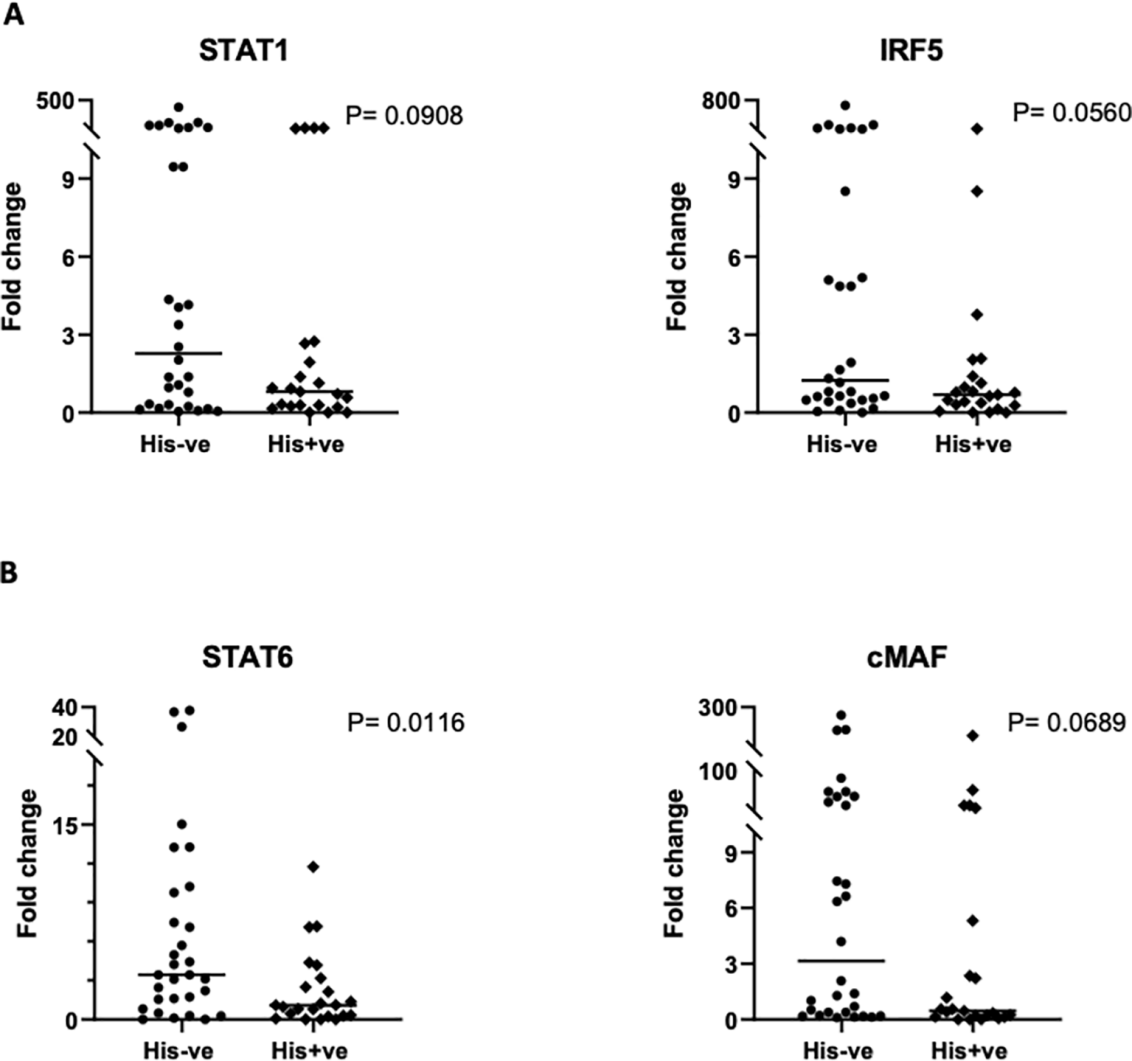
Comparison of transcription factor gene expression in placental macrophages based on qRT-PCR. **(A)** Analysis of *STAT-1* and *IRF5*, representing M1 transcription factors and, **(B)** analysis of *STAT-6* and *cMaf*, representing M2 transcription factors is illustrated. Data shown depicts the mean values of triplicates with values reported relative to the house-keeping gene, *GAPDH*. The 2ˆ (–delta delta CT) method was used for quantification of fold change. Statistical significance among the groups was assessed using Mann-Whitney U and student’s t-test.

**Figure 6:**
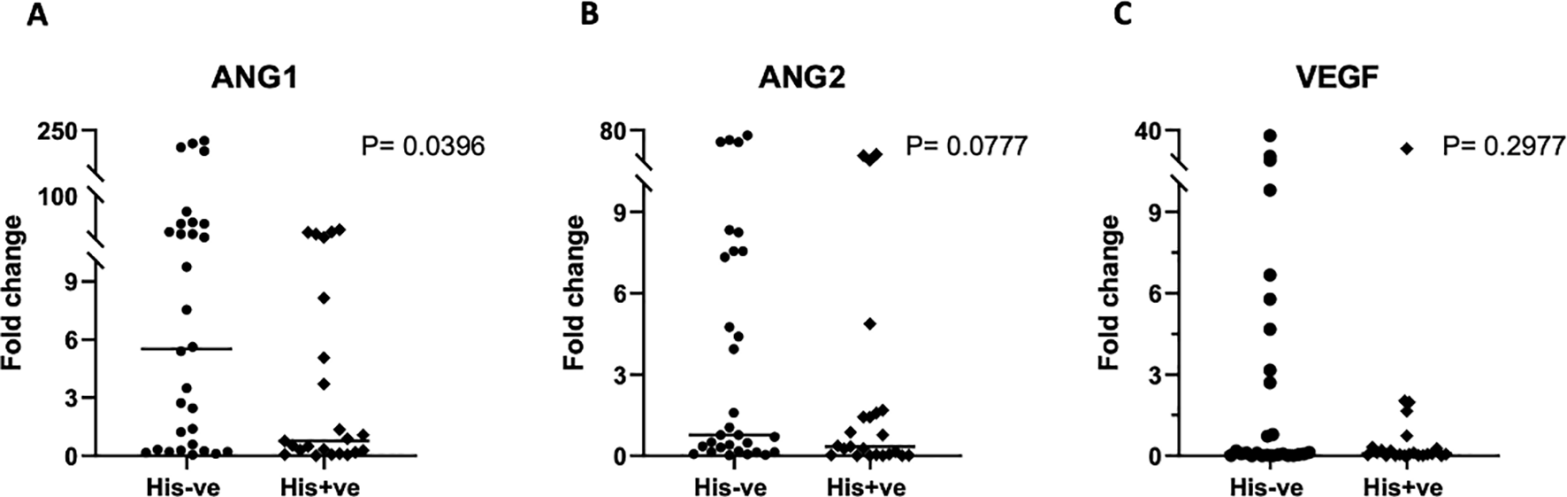
Gene expression analysis of angiogenic factors in placental macrophages. *ANG-1* **(A)** *ANG-2* **(B)** and *VEG*F **(C)** expression was determined by qRT-PCR, using samples ran in triplicates and the mean values used for quantification. All values are reported relative to the house-keeping gene GAPDH. To assess the significance in expression levels between the groups, Mann-Whitney U and student’s t-test were employed.

We also interrogated PM’s influence on the transcriptional regulation of macrophage activation, by examining the gene expression levels of key transcription factors. Real time PCR was used to quantify the relative expression of *STAT-1* and *IRF-5*, critical transcription factors for M1 polarization, and *STAT-6* and *c-Maf*, salient factors for M2 polarization, in placental macrophages, and compared between malaria positive and negative women. Variable levels of each factor were observed within each group (**Figure 5**). The expression levels of any given transcription factor except *STAT-6* did not differ significantly but had a trend toward decreased expression in malaria positive women. For *STAT-6*, there was a significant decrease (P = 0.0116) in malaria positive women compared to malaria negative women (**Figure 5B**), corroborating the role of this factor in pathogenesis of human malaria (38). Since angiogenesis is important for the development of placental vascular network, we further investigated how PM impacts the levels of angiogenic factors expressed in the decidual macrophages. Among angiogenic factors, the relative expression of *ANG-1*, *ANG-2* and *VEGF* (key mediators of angiogenesis) was quantified by RT-PCR (**Figure 6**). We observed generally low detectable relative expression levels of *VEGF* across malaria infection status and lower expression levels of *ANG-1* and *ANG-2* in malaria positive pregnancies than their negative counterparts, but only statistically significant in *ANG-1* (P=0.0396) (**Figure 6A**) Similar findings have been described in the systemic circulation of malaria infected pregnant women, in other populations (39).

### Sensitivity analysis

Given the low concordance observed between PCR and histology results (**Supplementary Table S2**), possibly due to differences in the sensitivity and specificity of the tests, the high rate of past infections detected in the participants by histological findings, the presence of submicroscopic infections, or the persistence of parasite DNA following clearance in cases of placental malaria (40, 41), our primary analyses were based on malaria diagnosis by histology. To address these discrepancies, alternative sensitivity analyses based on stratification by PCR, have been reported in **Supplementary Figures S3**-**S7**. The results were tallying with those generated using the primary analysis whereby the cell surface marker analysis (**Supplementary Figures S2**, **S3**), showed similar patterns and gene expression analysis (**Supplementary Figures S4**-**S6**), depicted trends toward simar patterns in the comparison of the different parameters with disease statusDiscussion.

The role of macrophages in the pathophysiology of pregnancy complications such as preeclampsia, gestational diabetes and villitis has been well documented (16, 42–44). However, there is a paucity of this information for other common complications including maternal infections like placental malaria. The main objective of this study was to characterize the functional phenotypes of macrophages in the decidua that may be associated with malaria infection in the placenta. To our knowledge, this is the first study evaluating the characteristics of placental macrophages in the context of placental malaria. The phenotypic, transcription, and angiogenic profiles of decidual macrophages was measured and compared between women who were positive for placental malaria infection and those who were not infected.

Placental infections can significantly impact pregnancy outcomes for both the mother and child since the placenta acts as a critical bridge between the mother and the developing fetus, mediating the transfer of nutrients, gases, and waste (45). An infected placenta triggers a host of inflammatory responses that may disrupt the normal immune balance consequently leading to poor placental function (2, 3, 46–48). It has been established that a major component of the inflammatory response to PM is the dense accumulation of intervillous inflammatory cells that mainly constitute macrophages (12, 47, 49). These macrophages are considered cardinal conveyors of protective immunity to the infection and have been shown to be effective in phagocytosing parasite derived material and contributing to parasite clearance (50, 51). It is thus conceivable that the local immune response is deviated from the anti-inflammatory pregnancy norm, toward a pro-inflammatory bias during PM infection impacting the adverse outcomes. A fine balance between protection from infection and protection from the negative effects of the pro-inflammatory response is paramount to ameliorate pregnancy outcomes during placental malaria infection. Macrophages, being one of the major players of the immune response to PM, present a unique cell population in that they have a distinct plasticity that confers them the capacity to exhibit opposing polarized functions (both pro-inflammatory and anti-inflammatory) which can be exploited to achieve this balance (52). It is thus important to understand the dynamics of macrophage characteristic features during placental malaria to decode how they can be manipulated and modulated to elicit favorable responses.

In this study of placental malaria in pregnant women living in a malaria endemic zone of western Kenya, infected and uninfected women had similar epidemiological and clinical characteristics. Gravidity, gestational age, newborn birth weight and hemoglobin levels were not significantly different between the two groups of women. Additionally, these variables did not appear to predict malaria infection in this cohort (**Supplementary Table S2**). These findings may be a positive reflection of Kenya’s IPTp policy which recommends provision of intermittent preventive treatment for malaria in pregnancy with sulfadoxine-pyrimethamine (IPTp-SP) during antenatal care visits in malaria-endemic zones (45) as illustrated by the similar mean dosage of IPTp between the infected and uninfected groups. Coupled with prompt diagnosis and effective treatment policies, these women might not experience the adverse consequences of pregnancy associated malaria. Intricately, when stratification of participants was based on malaria diagnosis by PCR, we observed that infected cases were significantly younger as is expected in high malaria transmission areas where women of lower reproductive age have low acquired immunity (53). While PCR is sensitive in detecting parasite nucleic acids, it does not distinguish those of a residual non-viable sequestered parasite origin, from those of a viable one (54).Moreover, histopathology analysis revealed that most infections detected were past infections due to the number of hemozoin pigment observed. Accordingly, for our study, comparison of inflammatory responses was premised on histology as it is the reference standard for placental malaria infection.

We observed that placental malaria did not influence an imbalance between pro- and anti-inflammatory macrophages based on the surface marker expression. Both single marker-(CD80 or CD86 and CD206 or CD163/CD209) and double marker- (CD80 +CD86 and CD206+CD163/CD209) expression employed for the dichotomy of M1 and M2 phenotype respectively, showed equal distribution of the cell populations between the infected and uninfected placentas. We posit that because the activation status of macrophages is a consequence of swift response to environmental cues, the timing of sampling, which can only happen after delivery, may misrepresent the earliest events in the infection process especially because majority of the infections in our study were past infections, as defined by the rationale for histological classifications of PM, previously reported (49). In this setting it is possible that after a heightened pro-inflammatory response during the active infection, with M1 macrophages being predominant (and the parasite density is lowered), an anti-inflammatory response with predominating M2 macrophage ensues in an attempt to restore the maternal-fetal tolerance that is essential to maintain pregnancy. This is illustrated by the significantly higher M2 macrophages, when compared to M1, independent of malaria infection status (**Supplementary Figure S2**). This hypothesis is consistent with prior reports on placental macrophages in symptomatic placental malaria (55) and monocytes/macrophages in asymptomatic peripheral malaria (56).

The molecular basis of the observed phenotypic features was further explored by analyzing the transcription regulatory pathways that may be involved in the maternal macrophage immune response. Comparison of the relative expression levels of the transcription factors; *STAT-1* and *IRF-5* (M1 macrophages) and *STAT-6* and *c-Maf* (M2 Macrophages), between PM-positive and PM-negative placentas showed a trend toward lower expression for both M1- and M2-macrophage associated factors in infected placentas, with only statistical significance in the levels of *STAT-6* expression. These data indicate that transcriptional regulation alone is not responsible for the phenotypic characterization of these macrophages and other factors including the nutritional status of these cells, re-infection history of participants and other factors associated with malaria transmission (eg microbiota composition, co-infection history), could play additional roles. Noteworthy, however, the significantly lower levels of *STAT-6* in the infected placentas could signify another importance of the *STAT-6* pathway as an essential player in IgE synthesis. IgE antibodies have been shown to have a pathologic role in malaria infection (57) and *STAT-6* has been implicated in malaria pathogenesis, shown to influence severity of the disease (38). Therefore, a decreased expression of this gene could be indicative of a hushed inflammatory response, consistent with our earlier observations.

Additionally, we assessed whether PM could have an impact on regulation of the angiogenic pathway in placental macrophages, essential for placental vascular development. Similar to findings reported by other researchers (39, 58, 59), our data demonstrate that the relative expression level of the key angiogenic factor *ANG-1* was significantly lower in malaria infected placentas. In areas of high malaria transmission, decreased levels of peripheral and placental *ANG-1* have been associated with detrimental effects including the occurrence of low birthweight (58). In our study, no association with birth weight was observed. There was no difference in the expression levels of *ANG-2* and *VEGF* between infected and uninfected placentas. The overall impact of PM in inflammatory and angiogenic pathways was relatively low in our study probably owing to the readily available IPTp-SP and diagnosis and treatment during the course of pregnancy as indicated by the large proportion of the infections being past infections.

A limitation of our study was its cross-sectional design, which prevented accurately assessing the participants’ medical histories, potentially introducing confounding factors. In addition, as an ex vivo study mainly characterizing the functional phenotypes of decidual macrophages we did not show functional responses associated with the observations and are thus limited in integrating the effects unequivocally. Future studies that take into account the whole set of variables related to malaria transmission and elucidate the functional consequence of the cellular subsets could provide a more definitive assessment. Furthermore, a more integrated approach consisting of the study of macrophage cell populations in malaria infected human placenta by single cell sequencing, in combination with other techniques like spatial transcriptomic that can allow characterization of gene signature of these cells in their local space and microenvironment could lead to more understanding of various interactions and mechanisms involved. To provide a comprehensive analysis of the effect of the stimulus from malaria infection on the transcription factors it could also be interesting to interrogate their phospho-activation states.

## Conclusion

This study underscores the critical role of placental macrophages in malaria infection, revealing previously underappreciated dysregulation in immune signatures and angiogenesis following placental malaria. While other placental immune cells may contribute to the disease, macrophages stand out as key players due to their plasticity, phagocytic capabilities, and potential as therapeutic targets. Our findings highlight the need for further research into macrophage-based strategies for managing placental malaria, such as modulating *STAT-6* expression to support pregnancy and boosting *ANG-1* to promote vascularization. By targeting these specific macrophage subsets, future therapeutic interventions may offer promising avenues for improving maternal and fetal outcomes in malaria-endemic regions.

## Data Availability

The original contributions presented in the study are included in the article/**Supplementary Material**. Further inquiries can be directed to the corresponding author.
