# Supplementary material for: Placental malaria infection is associated with downregulation of STAT-6 and ANG-1 in decidual macrophages
Source: Front Immunol. 2025 Feb 11;16:1497936. doi: 10.3389/fimmu.2025.1497936 (PMC11850307; doi:10.3389/fimmu.2025.1497936)
Supplement: Supplementary file 1 [file DataSheet1.pdf]

Placental Malaria Infection is Associated with Downregulation of STAT-6 and ANG-1 in Decidual Macrophages

Fred Owino[1, 2,3], Caroline Kijogi [3,4,5#*], Omu Anzala[6,7], Edwin Walong[8], Obiero Jael[9], Steven G. Nyanjom[1], Agola Lelo Eric[2], Bernard N. Kanoi[3,4], Jesse Gitaka[3,4]

[1]Biochemistry Department, Jomo Kenyatta University of Agriculture and Technology, Kenya.

[2]Kenya Medical Research Institute, Nairobi, Kenya.

[3]Centre for Malaria Elimination, Institute of Tropical Medicine, Mount Kenya University, Thika, Kenya.

[4]Centre for Research in Infectious Diseases, Mount Kenya University, Thika, Kenya

[5]Graduate School of Pharmaceutical Sciences, Tohoku University, Sendai, Japan

[6]KAVI Institute of Clinical Research, Nairobi, Kenya.

[7]University of Nairobi, Nairobi, Kenya,

[8]School of Medicine, Maseno University, Kisumu, Kenya

[9]Kenya Institute of Primate Research, Nairobi, Kenya

Shared first authorship, Caroline Kijogi

***** Corresponding author, Caroline Kijogi

Supplementary Figures and Tables

Supplementary Table 1: qPCR Primer list

Oligo Name	Oligo Seq	Modification
*ANG-1*_For	AAA TGG AAG GAA AAC ACA AGG AA	AAA TGG AAG GAA AAC ACA AGG AA
*ANG-1*_Rev	ATC TGC ACA GTC TCT AAA TGG T	ATC TGC ACA GTC TCT AAA TGG T
*ANG-2*_For	GAC GGC TGT GAT GAT AGA AAT AGG	GAC GGC TGT GAT GAT AGA AAT AGG
*ANG-2*_Rev	GAC TGT AGT TGG ATG ATG TGC TTG	GAC TGT AGT TGG ATG ATG TGC TTG
*c-Maf*_For	TGC ACT TCG ACG ACC GCT TCT C	TGC ACT TCG ACG ACC GCT TCT C
c-Maf _Rev	TGT ACA GCT CTC ACA CAA ATT TCA TTT TGT	TGT ACA GCT CTC ACA CAA ATT TCA TTT TGT
*GAPDH*_For	ACC ACA GTC CAT GCC ATC AC	ACC ACA GTC CAT GCC ATC AC
*GAPDH*_Rev	TCC ACC ACC CTG TTG CTG TA	TCC ACC ACC CTG TTG CTG TA
*IRF5*_For	GCC TGG GCC AAG GAG AC	GCC TGG GCC AAG GAG AC
*IRF5*_Rev	CCA CTT GGC CGG ATC G	CCA CTT GGC CGG ATC G
*STAT-1*_For	CCA TCC TTT GGT ACA ACA TGC	CCA TCC TTT GGT ACA ACA TGC
*STAT-1*_Rev	TGC ACA TGG TGG AGT CAG G	TGC ACA TGG TGG AGT CAG G
*STAT-6*_For	AGA GGG GTT GCC GAG GTG A	AGA GGG GTT GCC GAG GTG A
*STAT-6*_Rev	TGT CCA CCA GGC TTT CAC AC	TGT CCA CCA GGC TTT CAC AC
*VEGF*_For	CGA AGT GGT GAA GTT CAT G	CGA AGT GGT GAA GTT CAT G
*VEGF*_Rev	TTC TGT ATC AGT CTT TCC TGG TGA G	TTC TGT ATC AGT CTT TCC TGG TGA G

Supplementary Table 1; The qPCR primer sequences used for the detection of different genes including STAT-1, IRF-5, STA-6, and cMAF for transcription factors while ANG-1, ANG-2, and VEGF for angiogenic factors

Supplementary Table 2

Histology

PCR		+	-	TOTAL
	+	**12**	**11**	**23**
	-	**11**	**17**	**28**
	**TOTAL**	**23**	**28**	**<u>51</u>**

Key

1. Sensitivity = 52.17%
2. Specificity = 60.71%

Supplementary Table 2; PCR as an alternative placental malaria diagnostic method against the gold standard, histology, showed 52.17% sensitivity and as specificity of 60.71%.

Supplementary Figure 1

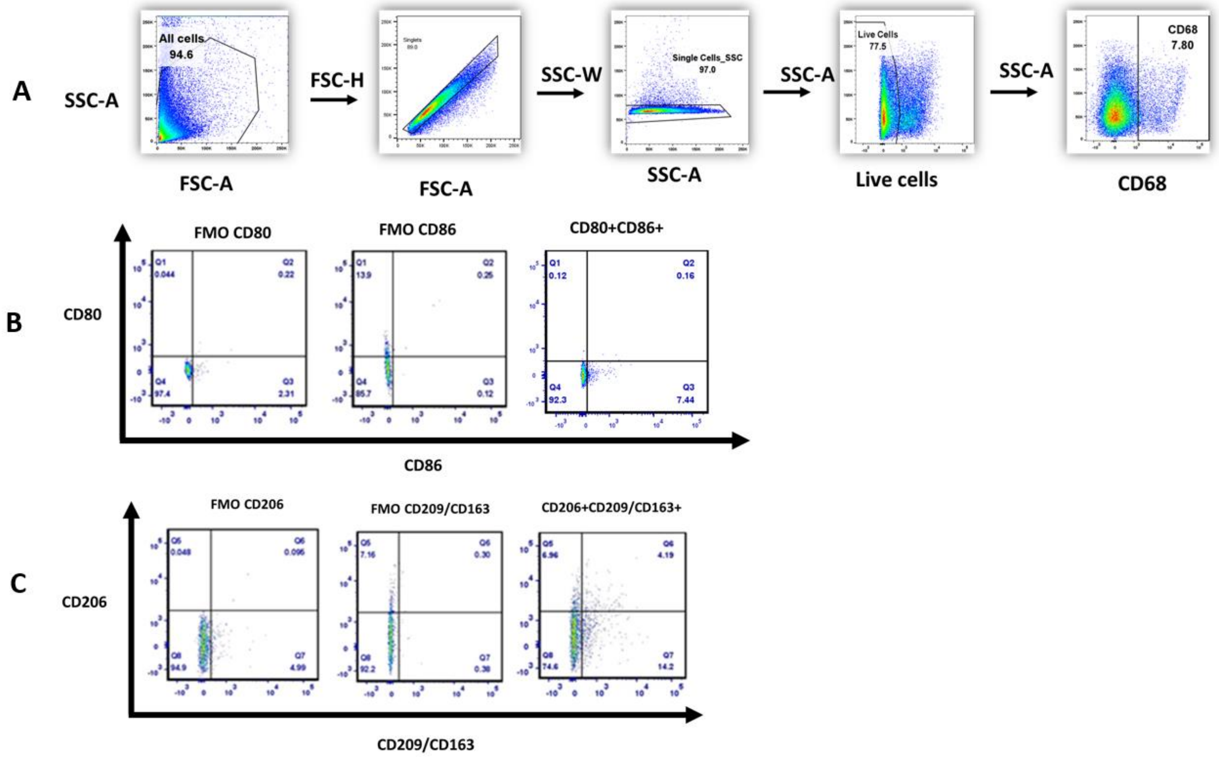

Supplementary_Figure 1; Representative dot plots depicting the gating strategy of the different markers used to define M1 and M2 macrophage subsets and their corresponding FMOS in flow cytometry analysis. Gating for the macrophage pan marker CD68, is illustrated in **A** while in **B** the FMOS for CD80, CD86, and full staining M1(CD80+CD86+) is displayed. **C** shows the FMOs for CD206/CD163, and CD209 representing the M2 subset.

Supplementary Figure 2

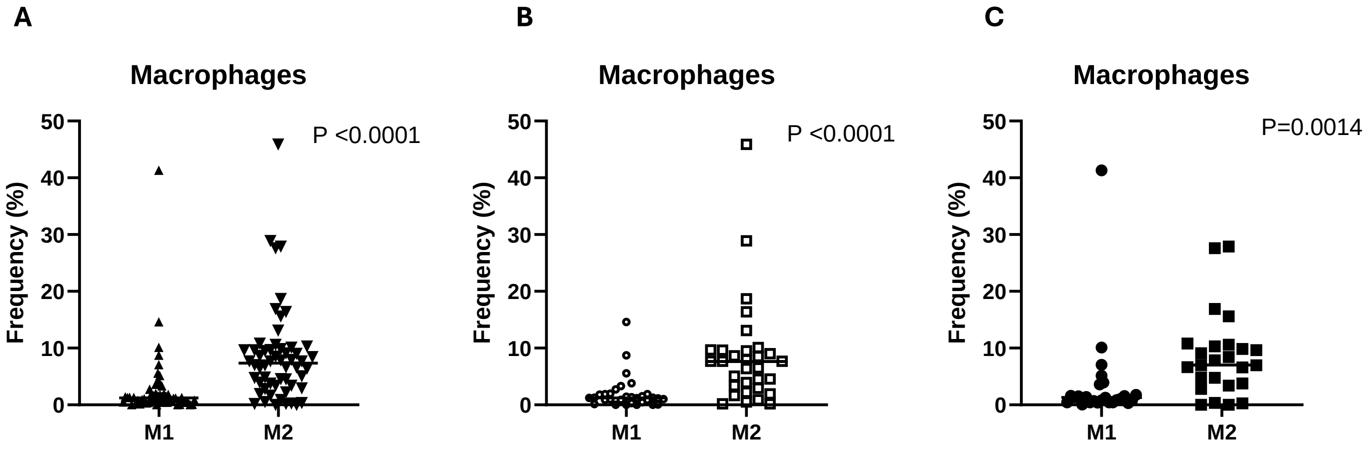

Supplementary Figure 2; Comparison of the distribution of M1 and M2 like macrophages from the placentas of all women **(A)** (P=0.0001), uninfected women only **(B)(**P=0.0001) and the infected women only **(C)** (P=0.0014) by Mann-Whitney U test.

Supplementary Figure 3

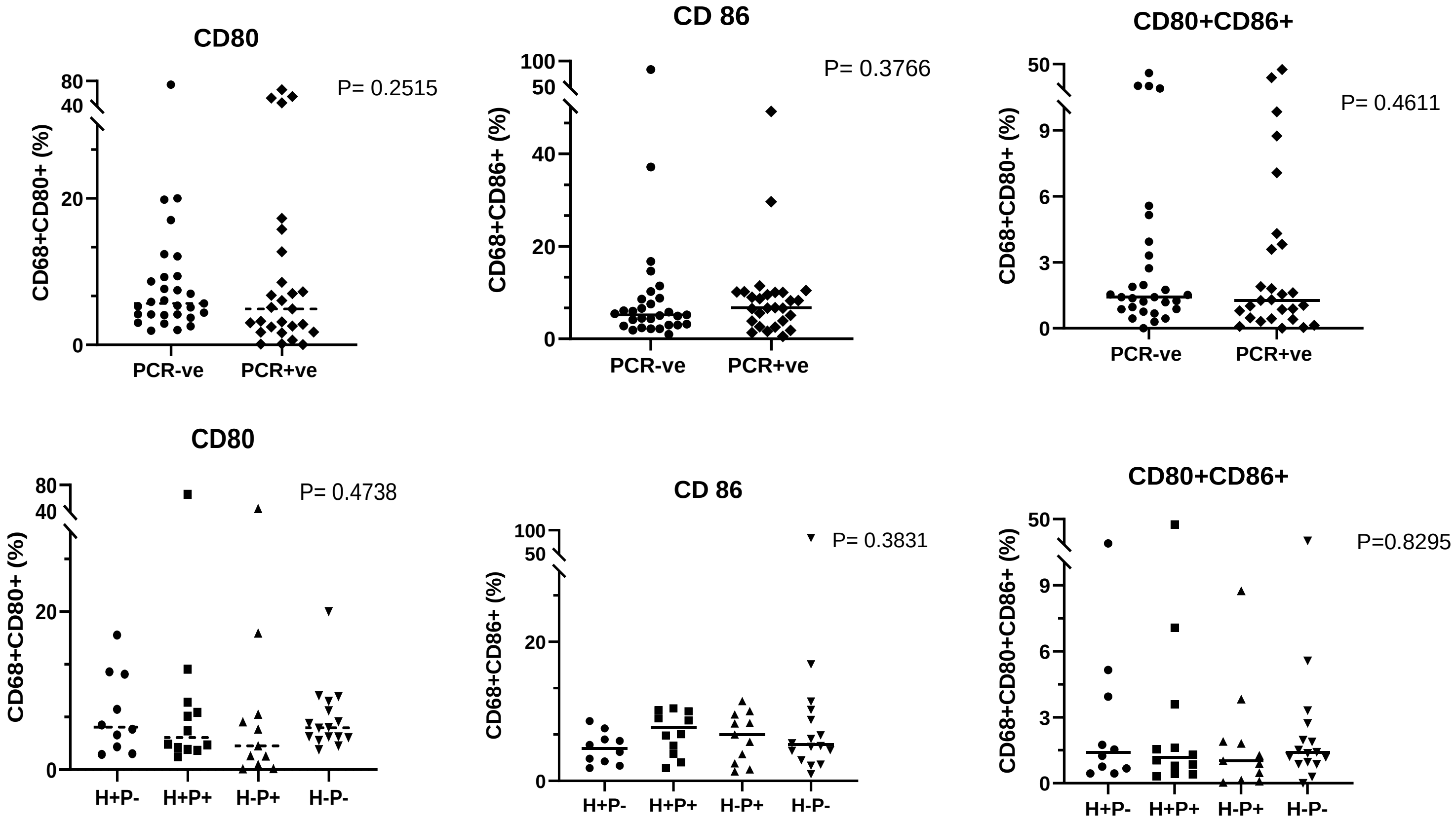

Supplementary Figure 3; Upper panel indicates analysis of M1 surface marker expression when samples were classified using PCR stratification. Lower panel: when stratification was by both histology and PCR. Mann-Whitney U test was used for pairwise compassions ANOVA and Kruskal- Walis were used for multiple comparisons.

Supplementary Figure 4

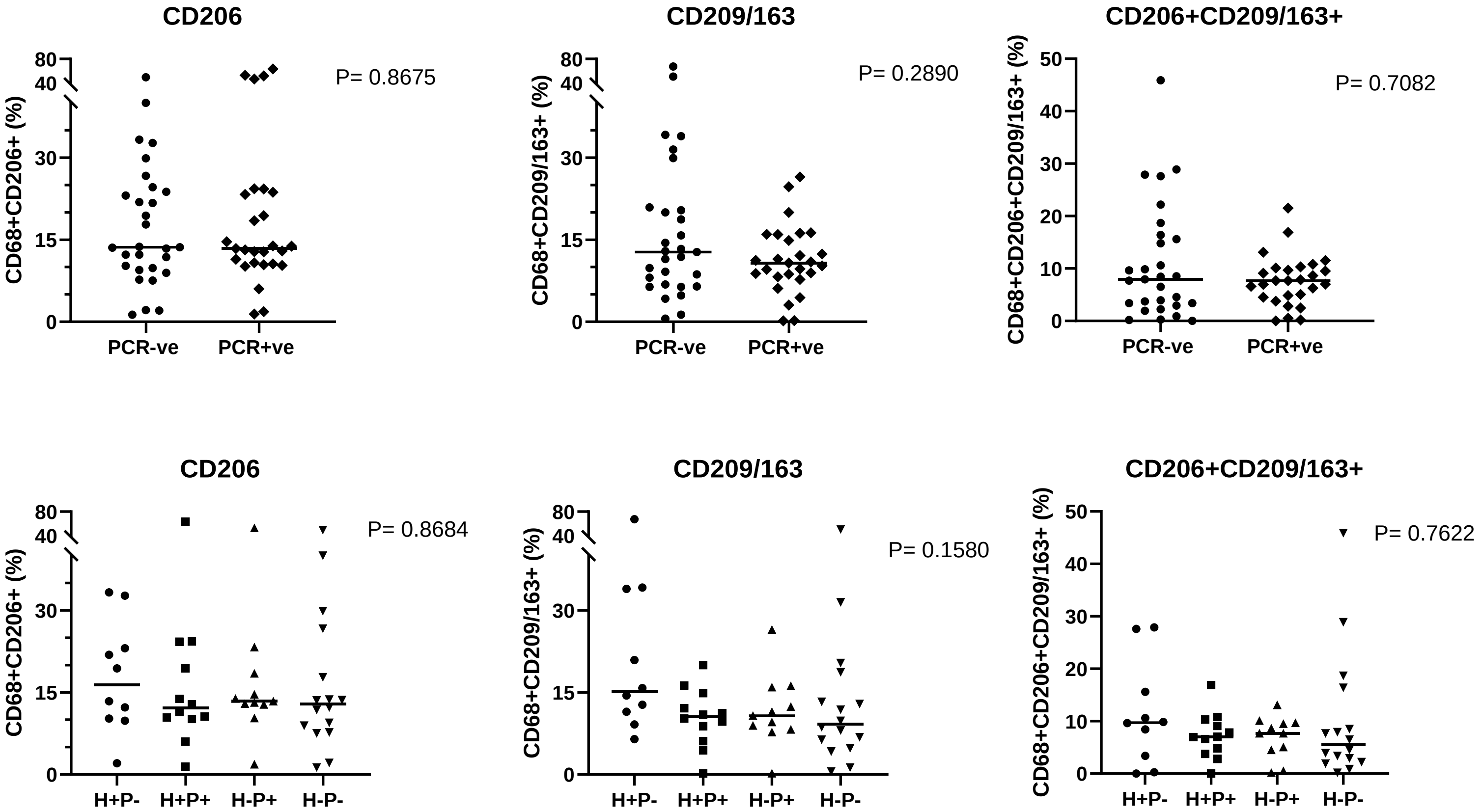

Supplementary Figure 4: Upper panel indicates analysis of M2 surface marker expression when samples were classified using PCR stratification. Lower panel: when stratification was by both histology and PCR. Mann-Whitney U test was used for pairwise compassions ANOVA and Kruskal- Walis were used for multiple comparisons.

Supplementary Figure 5

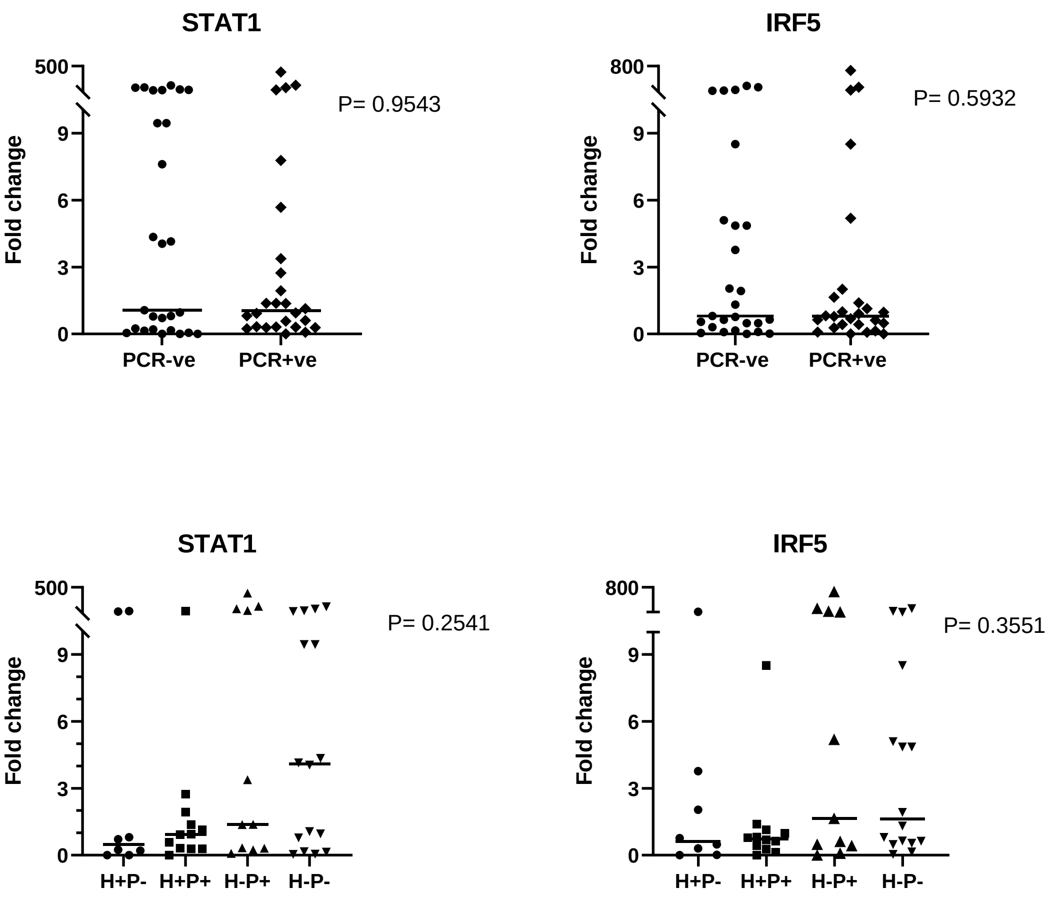

Supplementary Figure 5; Gene expression analysis of M1 transcription factors (*STAT-1* and *IRF-5*) as stratified by PCR (upper panel) and when stratification was by both histology and PCR (lower panel). Mann-Whitney U test was used for pairwise compassions ANOVA and Kruskal- Walis were used for multiple comparisons.

Supplementary Figure 6

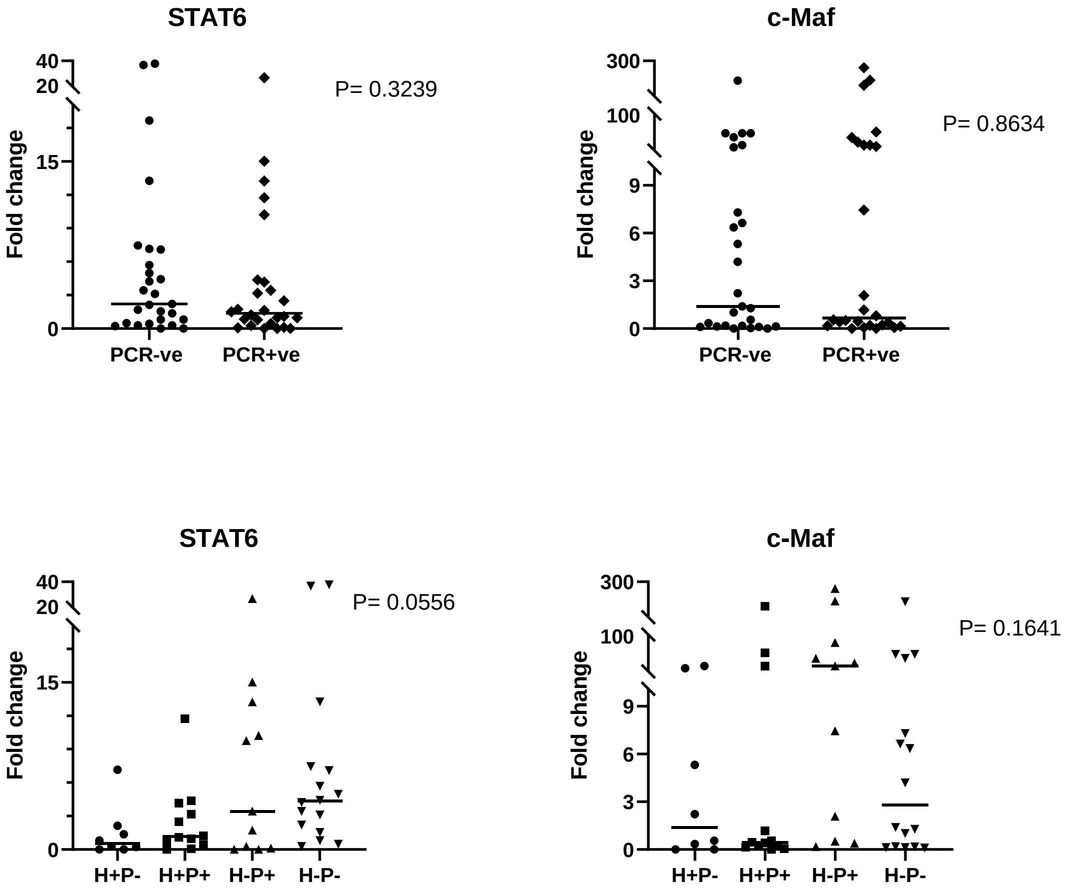

Supplementary Figure 6; Gene expression analysis of M2 transcription factors (*STAT-6* and *c-Maf*) as stratified by PCR (upper panel) and when stratification was by both histology and PCR (lower panel). Mann-Whitney U test was used for pairwise compassions ANOVA and Kruskal- Walis were used for multiple comparisons.

Supplementary Figure 7

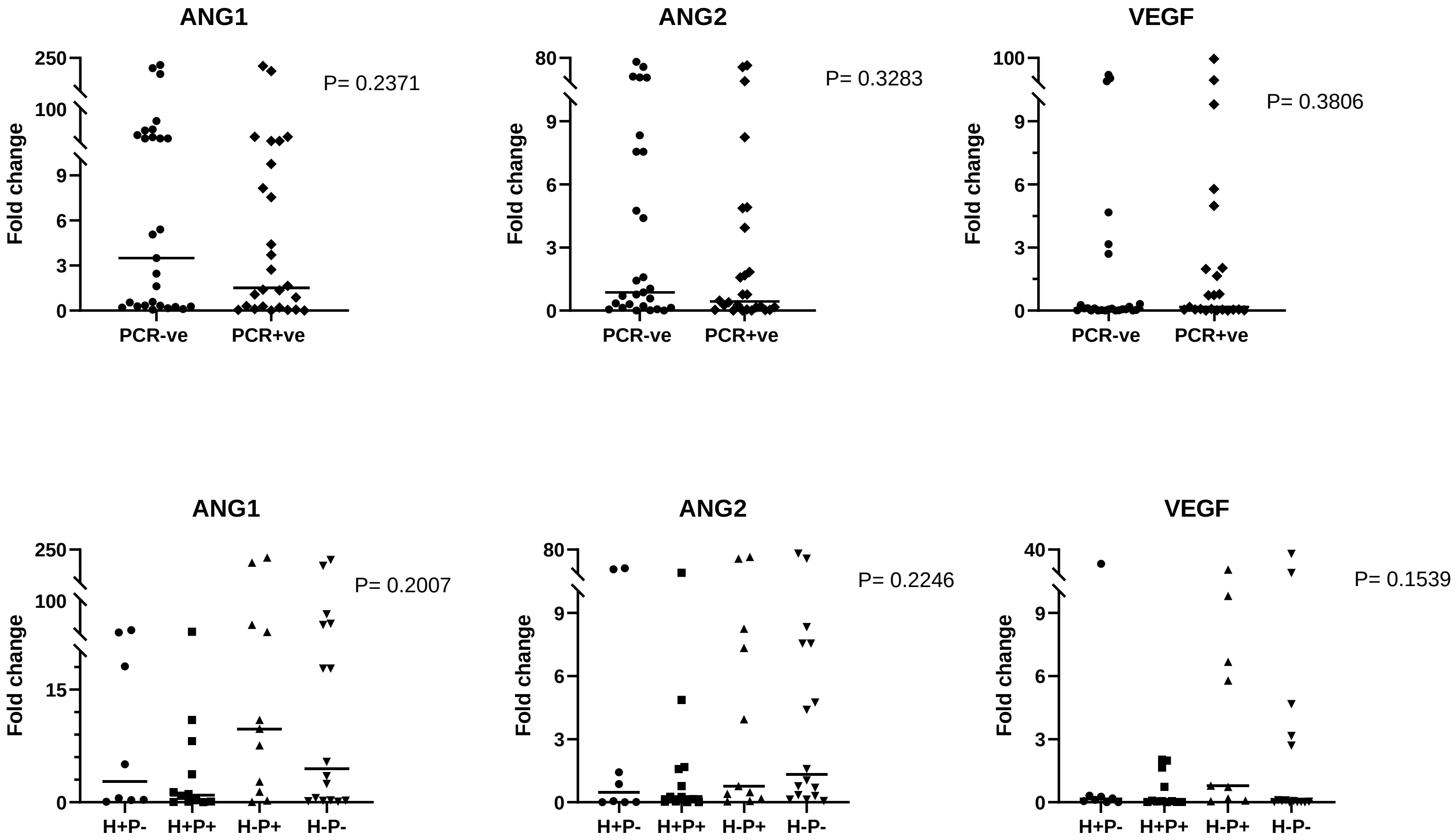

Supplementary Figure 7; Gene expression analysis of Angiogenic factors (*ANG-1*, *ANG-2* and *VEGF*) as stratified by PCR (upper panel) and when stratification was by both histology and PCR (lower panel). Mann-Whitney U test was used for pairwise compassions ANOVA and Kruskal- Walis were used for multiple comparisons.
